# A Quality Improvement Initiative to Improve Normothermia While Transitioning Premature Infants to an Open Crib

**DOI:** 10.1097/pq9.0000000000000809

**Published:** 2025-04-18

**Authors:** Nadia Campbell, Austin Han, Ranjith Kamity, Amrita Nayak

**Affiliations:** From the Department of Pediatrics, NYU Langone Hospital-Long Island, Mineola, N.Y.

## Abstract

**Introduction:**

Current literature focuses on the optimal lowest weight and incubator temperature to transition an infant to an open crib, with minimal data quantifying the rate of failed attempts or standardizing the process. Due to multiple failed attempts at this institution in 2021, the project aimed to reduce the rate of preterm newborns who failed the crib by 10% in 1 year.

**Methods:**

Interventions, including nursing education, an audit checklist, environmental changes, and a standardized protocol, were implemented after establishing baseline data.

**Results:**

The incidence of failed transitions to an open crib decreased from a baseline of 13.5% failed cribs per monthly transition attempts to 3.3% failed cribs per monthly transition attempts in 18 months, a 76% decrease, where it is currently sustained. Of note, infants born between 32 and 35 weeks gestation had higher failure rates compared with those born <32 weeks.

**Conclusions:**

Compliance with a thermoregulation protocol, utilizing an audit checklist, and standardizing the process improved the success rate of transitioning to an open crib.

## INTRODUCTION

One of the criteria for safely discharging infants from the hospital is maintaining core body temperature in an open crib. The infants’ adequate growth, development, and survival depend on this, as research has shown that achieving normothermia helps optimize outcomes and reduces mortality.^1^ Normothermia is defined as the body’s state of regulating a temperature ranging from 36.5 °C to 37.5 °C and depends on several factors, including infant weight, gestational age, infection, and environmental factors.^2^ According to the World Health Organization, hypothermia can be classified as mild (36.0 °C–36.4 °C), moderate (32.0 °C–35.9 °C), or severe (<32.0 °C).^3^ All neonates are at risk of hypothermia within the first few weeks of life, especially extremely premature and low birth weight infants, primarily due to evaporative heat loss from increased body surface area, increased skin permeability, and increased extracellular fluid.^4,5^ Newborn hypothermia can present as cold skin, pallor or acrocyanosis, tachypnea, lethargy, irritability, or poor feeding.^2^ Due to similarities in the presentation of isolated hypothermia, it is important to rule out other physiological or pathological causes.

This institution, NYU Langone Hospital-Long Island, has previously implemented policies aimed at minimizing the development of hypothermia in newborns in the delivery and operating room.^6,7^ Protocols and checklists are useful tools to standardize procedures, ensure adherence to current guidelines, reduce medical errors, and allow users to analyze trends in data.^8^ The institution did not have a standardized process for determining how long to monitor an infant’s temperature at the lowest incubator settings before transitioning.

Research is ongoing to establish clear guidelines and protocols to transition an infant out of an incubator; however, recommendations that predict suitable conditions for successful transitions are available.^9^ One study revealed that less than 50% of neonatal intensive care units (NICUs) had written guidelines. Those NICUs had different goals for transitioning criteria variables such as weight and temperature without 1 standardized protocol.^10,11^

Without standardized criteria for transitioning infants from an incubator to an open crib, it is challenging to assess the root cause for newborns who are unable to maintain normothermia in an open crib. Most studies commonly focused on the optimal lowest weight and incubator temperature to transition an infant to an open crib with minimal data to quantify the failure rate of preterm infants transitioning.^12–15^ Among moderately preterm neonates, studies have shown that transitioning from the incubator is associated with a higher velocity of weight gain when compared with staying in an incubator in infants transitioned at 1,600 versus 1,800 g.^16^ Studies also showed that NICUs implementing thermoregulation quality improvement (QI) projects significantly improved hypothermia rates.^17^

The SMART aim of this QI project was to reduce the rate of preterm infants failing the transition from an incubator to an open crib by 10% in 1 year. The global aim was to promote safe, timely, effective, equitable, and patient-centered discharge transition for newborns. The team used the SQUIRE 2.0 framework to describe the project.^18^

## METHODS

### Context

NYU Langone Hospital-Long Island is a tertiary care academic hospital located in the suburbs of Mineola, N.Y. The facility has a level 3, 36-bed NICU with approximately 5,000 live births and 700 annual admissions. On average, the institution has 355 preterm births annually, which is defined as births less than or equal to 36 weeks of gestational age.

### Establishment of Baseline Data

Before this project, the institution did not record data regarding the number of failed attempts to transition to an open crib. A failed attempt is defined as any infant not being able to maintain a body temperature of 36.5 °C for 48 hours once transitioned to an open crib. We noticed a high percentage of failed attempts in September 2021 and identified patient characteristics for the 7 failed attempts in September 2021. Upon further discovery, we found data from June 2021 showing similar rates of failed attempts. Six of the seven infants (85.7%) were born <35 weeks gestation and weighed <2,500 g at birth. Our June to September 2021 baseline rate was 13.5% before any interventions.

### Interventions

**Fig. 1.**
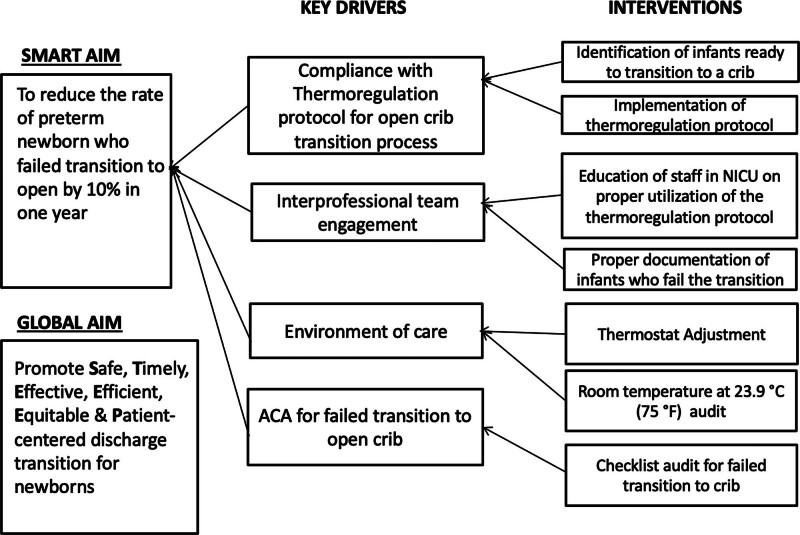
Key driver diagram detailing key drivers and interventions established for this QI project.

An interdisciplinary team of neonatologists, house staff, charge nurses, and other medical and nursing staff assembled to address the incidence of infants failing to transition from an incubator to an open crib. After obtaining buy-in from key stakeholders, the team decided to standardize the process of transitioning newborns to an open crib.^19^ A key driver diagram (Fig. 1) established goals and interventions.

### Measures

The outcome measure was the incidence of hypothermia (core temperature < 36.5 °C), resulting in a failed transition to crib per monthly total weaning attempts. The first process measure was compliance with the components of the failed crib audit checklist. This measure helped identify infants that had failed the transition and allowed further investigation into additional factors such as weight at the time of failure, corrected gestational age, and external environment temperature. The next process measure was maintaining the room temperature at 23.9 °C (75 °F). A third process measure was to check the compliance of infants remaining in an incubator for at least 24 hours once weaned to an incubator temperature of 27 °C. The balancing measures were hyperthermia (core temperature >37.5 °C), hospital readmissions for hypothermia after discharge from the NICU, and observed/expected length of stay (LOS). The observed/expected LOS was defined as the ratio of the mean duration of LOS for all cases to the mean of case-level expected LOS for all cases obtained from the institutional dashboard. This institution uses Vizient, a healthcare performance improvement company that provides data-driven solutions for healthcare improvement (Vizient, Irving, Tex.).^20^

### First Intervention: Nursing Education and Audit Checklist (Implemented October 2021)

**Fig. 2.**
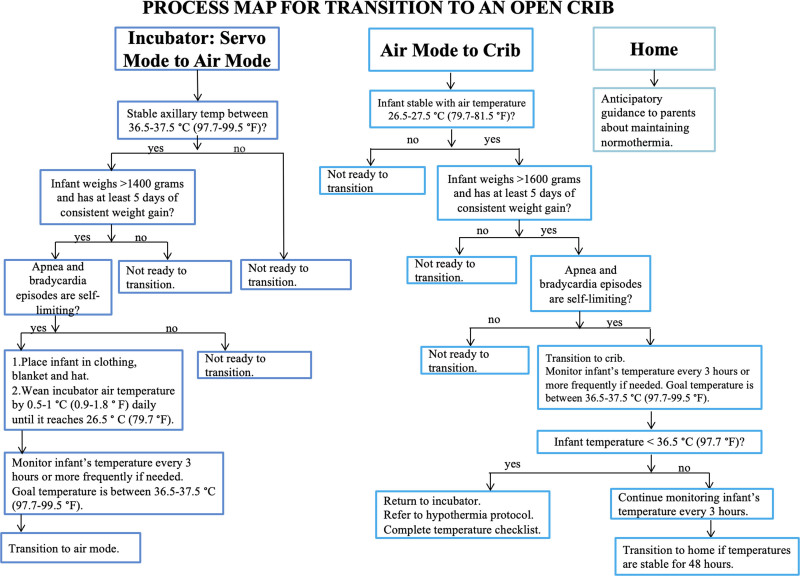
Process map utilizing NYU Langone Hospital-Long Island’s established thermoregulation protocol for transitioning preterm infants from an incubator to an open crib.

**Fig. 3.**
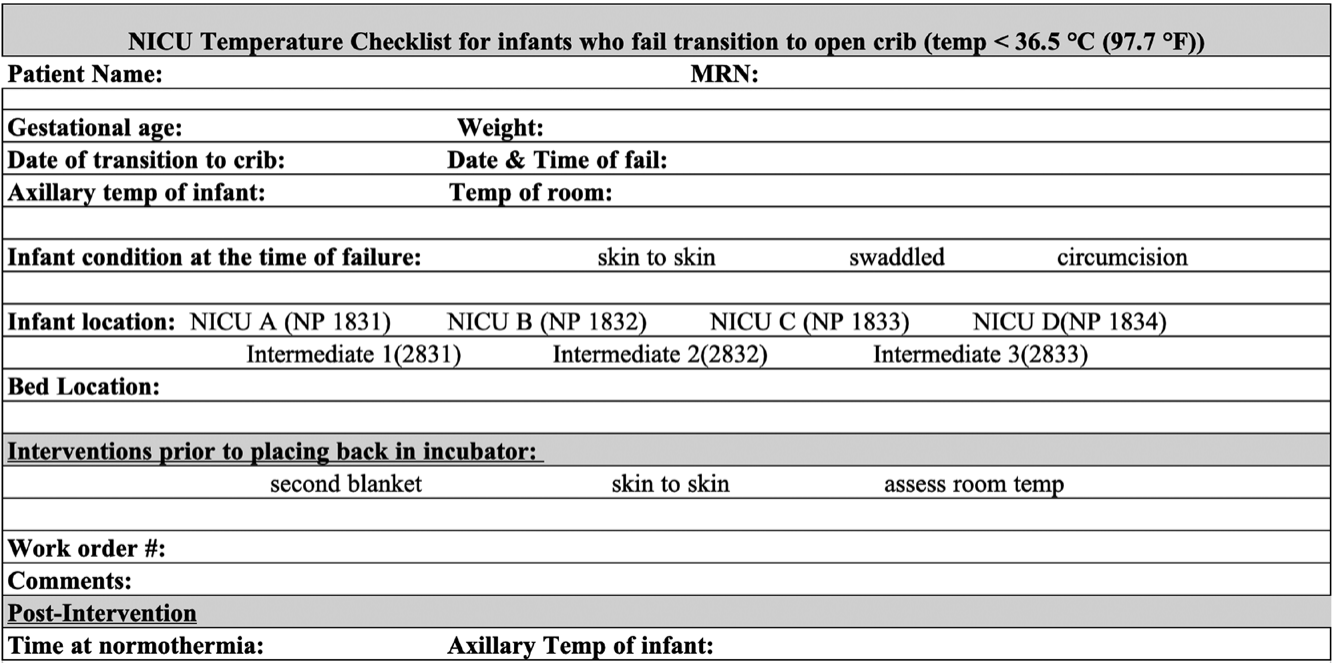
Failed crib audit checklist. An audit checklist to be completed by nursing staff if an infant fails the transition.

The first intervention involved educating nursing staff about the institution’s current thermoregulation protocol (Fig. 2) and the steps included in transitioning an infant from an incubator to an open crib; this included when to start the process, weaning the temperature of the incubator, monitoring the infant’s temperature before the transition, and ensuring the infant was appropriately dressed. The team created an audit checklist (Fig. 3) to be completed by the nursing staff if an infant failed the transition. The checklist included, but was not limited to, gestational age, weight at the time of failure, infant’s body temperature, temperature of the room, and interventions taken before returning the infant to the incubator. It also accounted for the degree of skin exposure, such as skin-to-skin with parents, swaddled, or undergoing a procedure.

### Second Intervention: Environmental Factor Adjustment (Implemented January 2022)

The team identified that environmental factors such as cold rooms could explain why infants failed to transition to an open crib. The American Academy of Pediatrics/American College of Obstetricians and Gynecologists recommends nursery/NICU environmental temperature ranges from 22 °C to 26 °C to help facilitate a safe transfer to a crib.^11^ In turn, with the assistance of the engineering department, the team set the thermostat in every room to 23.9 °C (75 °F).

### Third Intervention: Maintaining Normothermia in an Incubator Set to 27°C (Implemented May 2022)

To standardize the transition to an open crib, preterm infants <35 weeks gestation needed to maintain normothermia in an incubator set to 27 °C for at least 24 hours before transitioning to the crib. The prior practice was to maintain 27 °C without any standard duration of time. During this period, infants had axillary temperatures checked every 3 hours. Infants with temperatures lower than 36.5 °C, the lower end of normal, would remain in the incubator for 24 hours before the transition attempt.

### Fourth Intervention: Random Audits and Re-education (Implemented August 2022)

The QI team conducted random audits of procedure and checklist compliance and room temperatures, whereas the engineering department also conducted room temperature audits independently. Charge nurses also re-educated nursing staff in monthly multidisciplinary huddles to reinforce safe transitioning practices and proper documentation of failed transitions.

### Study of Interventions

PDSA cycles were conducted every few months to roll out interventions and improve staff engagement. The trends were reviewed and correlated to interventions to determine if interventions had the intended effect. The team also analyzed completed audit checklists and engaged with frontline staff using unit huddles to ensure that previous interventions were being done correctly, such as completing the checklist when there were failed weans. Engineering controls were followed to show compliance with the goal temperature in the NICU rooms. Initially, random spot checks monitored temperatures in the rooms; however, beginning in January 2023, continuous monitoring was done with daily spot checks. We also instituted an incident reporting system to capture failed compliance with the third intervention (maintaining temperatures in the incubator for 24 hours before weaning to the crib). PDSA cycles were crucial to assessing the effectiveness of the interventions and keeping staff engaged.

### Analysis

This project used statistical process control charts (P chart) to follow the proportion of failed cribs per total monthly transition attempts displayed over time in months. The team designated a process change in October 2021 to mark the start of the study. The team used Institute for Healthcare Improvement healthcare control chart rules for stability analysis.^21^ The team created statistical process control charts and performed statistical analyses using the QI Macros plug-in (KnowWare International, Denver, Colo.) on Microsoft Excel 365, version 2410 (Microsoft Corporation, Seattle, Wash.).

### Ethical Considerations

This QI initiative had no ethical concerns or conflicts of interest. This project met the NYU Langone Hospital-Long Island institutional review board criteria as a QI initiative.

## RESULTS

**Fig. 4.**
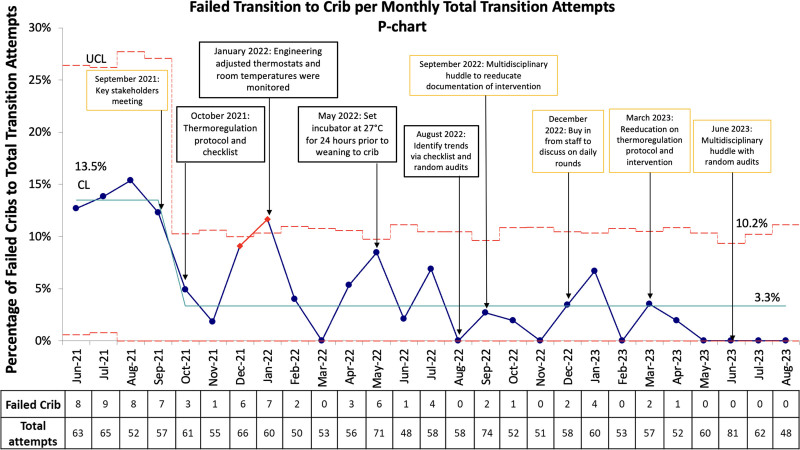
Statistical process control chart. P chart depicting the percentage of failed crib transitions per total monthly transition attempts: The *x* axis shows time in months. The data table below each month shows the number of failed cribs and the total attempts for that month. The *y* axis shows the percentage of failed cribs per total monthly transition attempts. The chart has interventions annotated. October 2021 marks a process change to designate the start of the interventions. Annotations in black represent major interventions. The baseline centerline shows 13.5% failed cribs/monthly transition attempts. The control chart reflects the process remaining in control from October 2021 to August 2023 around new CL at 3.3% failed cribs/monthly transition attempts. Special cause variations were noted in December 2021 and January 2022. CL, centerline; UCL, upper control line.

After the first intervention, nursing education and generation of the audit checklist in October 2021, the new centerline was established at 3.3% compared with the baseline of 13.5% failed cribs per monthly total transition attempts. Following initial improvement, we saw an increase in failed cribs during December 2021 and January 2022. The team implemented the second intervention of setting the thermostat in each NICU room to 23.9 °C (75 °F) in January 2022 and the third intervention of setting the incubator to 27 °C for a minimum of 24 hours before transitioning to an open crib in May 2022. The centerline remained stable from October 2021 to August 2023 without upward trends. After establishing the new centerline, subsequent steps (multidisciplinary huddles, reeducation of protocols, and random audits of room temperature and transitioning to an open crib) to reinforce the above interventions sustained the centerline at 3.3% failed cribs per monthly total transition attempts, with the process remaining in control (Fig. 4).

Postintervention, of the infants who failed the transition to a crib, 39 of 45 were preterm. Of those 39 preterm infants, 34 were born between 32 and 35 weeks gestation, and 5 were born <32 weeks. At the time of failure, 41 of 45 had a postmenstrual age of 33–36 weeks gestation. Thirty-seven (of 45) infants who failed were born weighing <2,500 g, with an average weight of 2,180 g at the time of failure (range 1,725–2,800 g).

Audit checklist compliance for this QI project, which is defined as the compliance with completing the audit checklist for every failed wean and calculated using the number of completed checklists for infants with documented failed transitions divided by the total number of infants known to have failed, was 75.6% (34/45). All infants with completed checklists met the full criteria for transitioning to an open crib. The team identified zero incident reports for noncompliance with setting the incubator to 27 °C for 24 hours before weaning from June 2022 to August 2023.

**Fig. 5.**
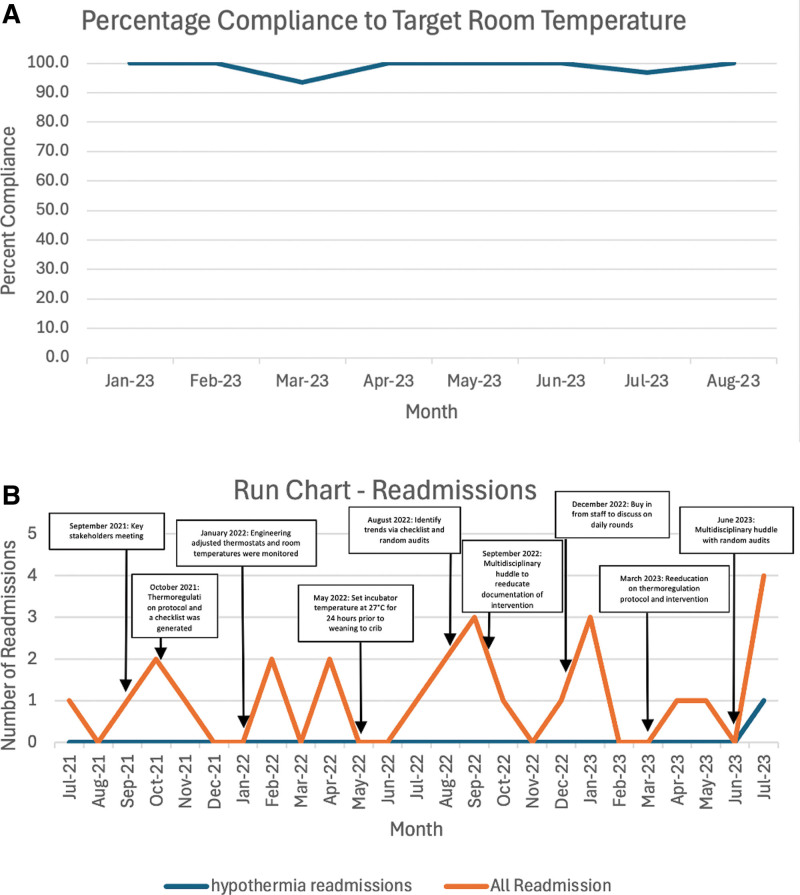
Process and balancing measure run charts. A, Run chart showing the percentage compliance of daily audits to the target room temperature of 23.9 °C (75 °F). The *x* axis shows the month recorded. The *y* axis shows the compliance percentage to the goal temperature during the month. B, Run chart showing the readmissions after NICU discharge. The orange line reflects all readmissions to the institution, whereas the blue line reveals the readmissions secondary to hypothermia. The run chart is also annotated with the time of each intervention implementation. The *x* axis is the month recorded. The *y* axis is the number of readmissions.

The random daily room temperature audits from January 2023 to August 2023 showed that the average compliance with the goal temperature was 93%–100% (Fig. 5A).

For the balancing measures of hyperthermia and observed/expected LOS, there were no infants with hyperthermia (N = 0/45) or increase in the LOS (observed/expected) from the fiscal year 2021 to the present. The observed/expected LOS in the NICU, automated data provided through the electronic medical records, decreased from 0.64 to 0.59 from 2021 to 2023. Moreover, our readmission rate for hypothermia was 3.8% (1/26) of all readmissions (Fig. 5B).

## DISCUSSION

### Key Findings

This QI project aimed to standardize the transition process and identify and address various causes that could lead to infants failing the transition from an incubator to an open crib. After several meetings and interventions with the QI team, the improved weaning-to-crib process decreased the incidence of infants failing the transition.

### Interpretation

After establishing the baseline rate of newborns failing the transition, this QI project implemented several interventions. The first intervention was an audit checklist with staff education about the thermoregulation protocol, which helped analyze the factors contributing to failed crib transition. Subsequent interventions were focused on optimizing environmental factors and standardizing the practice of transitioning to a crib. The second intervention was to set each NICU room’s thermostat to 23.9 °C (75 °F) to control the environmental factors. To ensure that infants were weaned only when ready, they were maintained in an incubator set to 27 °C for a minimum of 24 hours before transitioning to an open crib.

Initially, there was a decline in the number of failed transitions, followed by a gradual increase. The special cause variations in December 2021 and January 2022 warranted further investigation by auditing completed checklists and finding unaddressed causes for the increasing number of failed wean attempts, such as inconsistent external and borderline failing temperatures. Engineering controls were instituted to ensure compliance with desired environmental temperatures, followed by policy changes and increased frontline engagement using audits. With each intervention, the peak in rise in failed transitions became smaller. Process measures were also studied to ensure compliance with set interventions. Ultimately, the number of failed cribs per monthly attempt throughout the project decreased significantly. The control chart of the data showed the process remained in control, sustained around the new lower centerline.

### Strengths and Limitations

One of the strengths of this QI project was identifying the importance of frequently monitoring an infant’s temperature 24 hours before transitioning to an open crib to assess for adequate thermoregulation ability and readiness to wean. On several occasions, an infant had borderline temperatures warranting delayed transitioning. Another strength was using structural interventions to optimize external factors, such as maintaining set room temperatures to help infants maintain normothermia.

Although this initiative cannot assume sole responsibility for the decline in observed versus expected LOS due to multiple other factors, the interventions to improve thermoregulation did not have a negative effect on LOS. Further looking at the readmission rate throughout the project, there was only 1 case of hypothermia readmission out of the 26 infants readmitted postdischarge. Interestingly, this infant was not hypothermic during the initial hospitalization.

Curiously, most infants who failed the transition were born at 32–35 weeks gestation with a corrected gestational age between 33 and 36 weeks at the time of failure. It is unclear why this subgroup of infants had the most failures. It is plausible that infants born <32 weeks gestation had more time in an incubator due to their lower gestational age and birth weight. The protocol may tend to be emphasized more for infants born <32 weeks. Nevertheless, this observation allowed us to infer that the 32–35 weeks gestation preterm infants were impacted the most by this initiative and would likely benefit from further interventions and initiatives.

One limitation was the minimal baseline data collected at the beginning of the project. Of the 4 months of baseline data, the first three months preceding the intervention revealed higher numbers than the September 2021 baseline. However, patient characteristics were not captured systematically. This QI initiative was intended to improve the rates of failed crib weans over time. Other limitations include the infrequent occurrences and limited data of failed transitions. The data included 77 infants with failed transitions between June 2021 and April 2023; of them, 32 had failed before the implementation of the audit checklist, but the failure could not be captured due to no formal documentation process before the project initiation. The biggest limitation is the audit checklist compliance and documentation of interventions. Checklist compliance was crucial because patient characteristics were captured on the completed checklists. Therefore, when infants failed the transition and did not have a completed checklist, it was difficult to gather information such as corrected gestational age, weight, temperature at the time of failure and interventions taken before placing the infant back in the incubator.

In addition to hyperthermia and LOS as balancing measures, readmission rates are useful for determining discharge readiness. We found that only one infant was readmitted for hypothermia from all the readmissions documented, showing that our interventions for crib readiness were effective.

## CONCLUSIONS

Although underlying medical conditions will continue to contribute to infants failing to maintain normothermia, this QI initiative aimed to create a structured approach to mitigate external factors for hypothermia, thereby reducing unnecessary delays in hospital discharge and promoting safe, timely, effective, equitable and patient-centered discharge transition for newborns. The team assessed the common trends and geared interventions directly for these infants. This project highlights that the highest failure rates were in the 32–35 weeks gestational age group, and future considerations warrant interventions targeted to this subgroup of infants. The electronic medical record system will account for missed compliance with the audit checklist and enforce the importance of timely documentation of failed transitions. Reinforcing the current thermoregulation protocol and implementing an audit checklist helped standardize the transition to an open crib, heighten awareness, and improve the success rate of transitioning infants to an open crib.
